# Does Application of Lymphatic Drainage with Kinesiology Taping Have Any Effect on the Extent of Edema and Range of Motion in Early Postoperative Recovery following Primary Endoprosthetics of the Knee Joint?

**DOI:** 10.3390/jcm11123456

**Published:** 2022-06-16

**Authors:** Magdalena Sobiech, Agata Czępińska, Grzegorz Zieliński, Magdalena Zawadka, Piotr Gawda

**Affiliations:** Department of Sports Medicine, Faculty of Health Sciences, Medical University of Lublin, 20-093 Lublin, Poland; magdalena.sobiech@umlub.pl (M.S.); grzegorz.zielinski@umlub.pl (G.Z.); magdalena.zawadka@umlub.pl (M.Z.); piotr.gawda@umlub.pl (P.G.)

**Keywords:** osteoarthritis, rehabilitation, swelling, total knee arthroplasty

## Abstract

Background: The surgery of knee replacement due to degenerative changes is the last step of the treatment. After surgery, a major problem in patients is pain, swelling, intraarticular hematoma, and the restriction of the mobility of the joint. The aim of this work was to determine the effect of Kinesio Taping (KT) on reducing edema of the subcutaneous tissue and improving the range of motion in the joint. Methods: 82 patients were qualified for the study. After surgery, 42 patients received postoperative edema treatment with KT bands, and 40 patients did not receive the treatment. The swelling thickness and range of mobility were measured on the third and eighth days after the operation. Results: A statistical difference between the longitudinal measurements of the KT group and the group without KT application was shown at the level of the fibula head, 25 mm below the fibula neck, and 50 mm below the fibular neck. There were no statistically significant differences in the change in knee angle between the applied and non-applied patients. Conclusion: The lymphatic application technique KT influences the absorption of subcutaneous edema after primary knee joint replacement surgery but has no influence on mobility.

## 1. Introduction

Osteoarthritis (OA) is a modern-age disease of an unknown etiology and a process including various types of factors, such as genetic, metabolic, inflammatory, and mechanical factors [1,2,3]. According to specialists, the symptoms of osteoarthritis are revealed in X-Ray imaging in one-third of the world population [4]. Regarding etiology, osteoarthritis can be divided into two types: primary (idiopathic) of unknown etiology and secondary [5]. A knee joint is one of the most vulnerable to overload and the occurrence of degenerative lesions, so-called gonarthrosis [6]. It is related to its structure and the function it serves in transferring heavy loads [7]. The symptoms of gonarthrosis are characterized by pain at the initial stage of motion, flexion deformity of the affected joints, morning stiffness lasting up to 30 min or stiffness after inactivity, crepitus occurring during movement, and joint effusion [8]. In the next stage of the disease, outline thickening and joint instability occur [9]. Conservative treatment of osteoarthritis is based on pain relief, flexion deformity reduction, muscle strength retention preventing joint contractures, tissue swelling reduction, and gait function improvement [10]. Conservative treatment also includes physiotherapy and pharmacological treatment [11]. Surgical treatment of a knee joint is the last part of osteoarthritis treatment. Knee endoprosthesis is a surgical procedure to replace the damaged joint structure with new elements [12]. An artificial knee joint is made of metal elements that imitate distal femoral epiphysis and proximal tibial epiphysis [3,12,13]. They are separated with a spacer made of plastic. The method of surgery and the choice of implant depend on many factors, i.e., degenerative changes in a knee joint, the age of a patient, and their general condition [12,13]. In the first few days after the surgery, a patient may be affected with the following problems: the pain of an operated limb, intra-articular hematoma, subcutaneous edema, and flexion deformity. Long-lasting symptoms may inhibit the process of post-surgical and full recovery [14]. Regarding the growing number of patients undergoing surgery as a result of advanced osteoarthritis, it is legitimate to seek new and effective physiotherapeutic methods applied in the initial phase of a postoperational period) [15,16]. One of the most advanced therapies supporting postoperational recovery, which functions 24 h a day, is the method of dynamic application of Kinesio Taping (KT) [15,16]. Therapeutic knee patching combined with moderate adapted training has been proven to be an effective method for managing pain and disability limitations in patients with knee osteoarthritis [17]. According to the author of this method, it is possible to achieve pain-relieving, anti-swell, corrective, and sensation-improving effects, depending on different types of tensions and applications of the tape [16]. The applied KT lymphatic technique is based on the application of the tape with the tension of 15% with the anchor near the proximal lymph node [15,18,19,20]. Its aim is to enable the flow of lymph and blood from the place of congestion and lymphedema by a slight elevation of the skin. Tape properties, such as wave pattern, elasticity, thickness, and weight, similar to skin parameters, enable the extension of the space between the dermis and fascia, which improves the uninhibited lymph flow [15,18,19,20]. A proper application also enables the normalization of muscle and fascial tonus and their corrective positioning. It results in muscle loosening and supports its work during the motion. KT assumes the application of natural self-healing processes of the organism [15,18,19,20]. The advantage of this method is safety and the low cost of the therapy [21].

On the basis of the above considerations, it can be assumed that in patients who underwent the primary surgery of knee endoprosthetics, the application of the KT lymphatic drainage technique will enhance the subcutaneous edema resorption and improve the range of motion (ROM) in a knee joint in the first days after the surgery.

## 2. Materials and Methods

In order to obtain a homogenous group, patients qualified for the revision surgery of knee endoprosthetics, patients who suffered or are currently suffering from deep vein thrombosis of the lower limbs, patients qualified for the primary endoprosthetics as a result of post-traumatic osteoarthritis, and patients, after other surgeries carried out on the lower limbs, were excluded from the research. The criterion for entering the group was the qualification for the primary endoprosthetics of a knee joint on the basis of clinical symptoms according to the American College of Rheumatology (ACR) and radiological symptoms according to Kellgren–Lawrence Scale.

After the application of the above criteria, 82 inpatients (17 males and 65 females, median age 67.22 ± 7.14) of the Chair and Department of Rehabilitation and Orthopaedics and Chair and Department of Orthopaedics and Traumatology of the Independent Public Teaching Hospital No.4 in Lublin were qualified, in the period from October 2018 to March 2020 (Table 1). Each qualified patient was provided with an information sheet, and after examining the scope of research, they signed informed consent for participation in the research. The patients were informed about the possibility of withdrawing at any stage of the research. The Bioethical Commission operating at the Medical University of Lublin issued consent for the research to be carried out (Resolution of Ethical Commission, no. KE-0254/288/2018). The group of patients who qualified for the research was divided into two subgroups: a group of 42 members where, in the third 24 h after the surgery, the lymphatic application of Kinesio Taping was introduced into the postoperative edema treatment; and a group of 40 members where dynamic tapes were not applied.

### 2.1. Methods

#### 2.1.1. Clinical Assessment of Disease Activity in Osteoarthritis

The Kellgren–Lawrence scale was applied for the clinical assessment of disease activity in osteoarthritis. It is a five-step grading system allowing for the assessment of radiological changes in patients with osteoarthritis. The radiological assessment of the disease activity was based on radiograms in the anterior-posterior (AP) view that revealed the presence of osteophytes, joint gap narrowing, and subchondral bone sclerosis [22,23]. Patients graded III and IV on the Kellgren–Lawrence scale were qualified for the research.

#### 2.1.2. Assessment of Postoperative Edema

**Figure 1:**
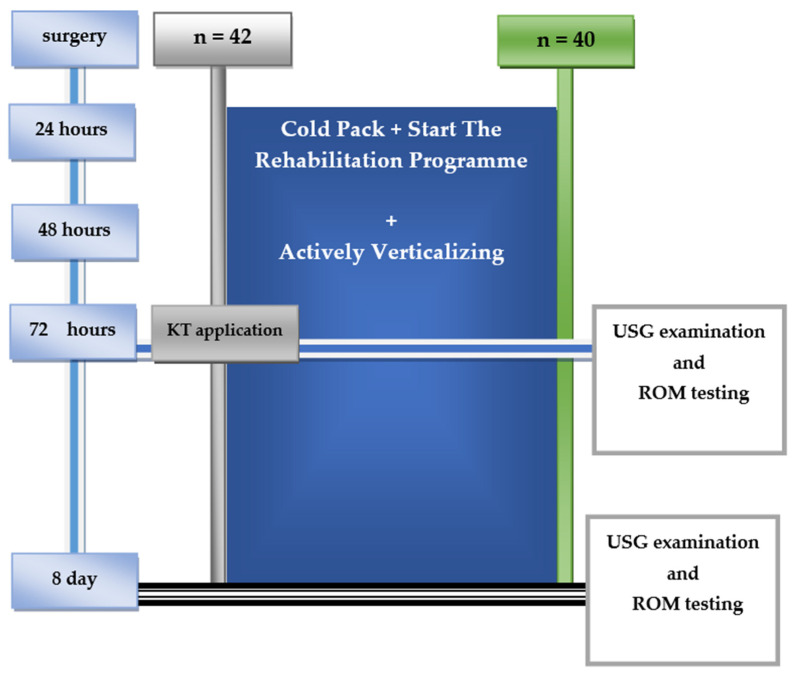
Demonstration of the conduct of a time-divided experiment. ROM—range of motion; KT—Kinesio Taping; USG—ultrasound.

The measurements of edema thickness in the proximity of a knee joint above the fibula were carried out in both groups of patients in the third and eighth 24 h after the surgery by a Simens ACUSON S2000 HELX EVOLUTION (Siemens, Erlangen, Germany) with a linear probe, 18L6 HD. Measurements were taken at the level of the fibula head, 25 mm below the fibular neck, and 50 mm below the fibular neck along the longitudinal axis (Figure 1).

#### 2.1.3. Application of Dynamic Kinesio Tape

**Figure 2:**
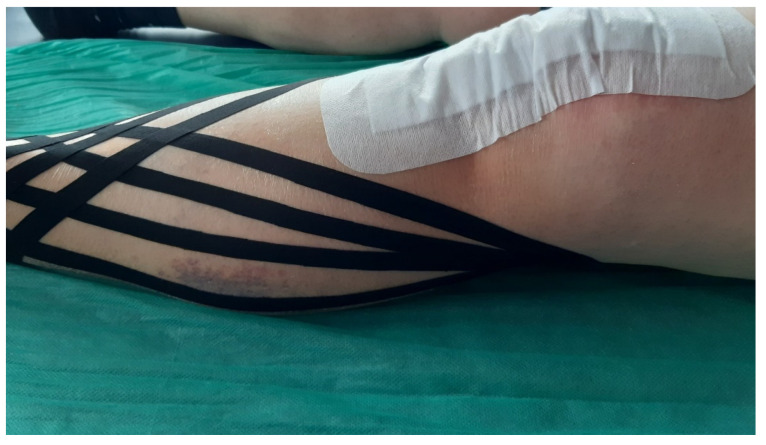
KT Lymphatic application on the lower leg.

**Figure 3:**
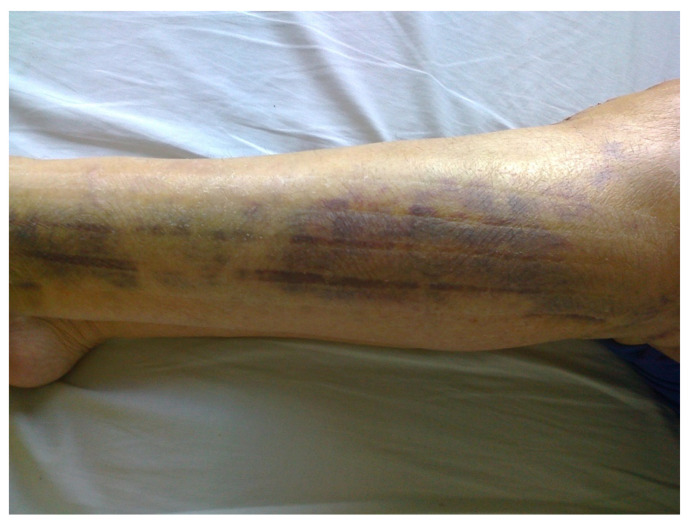
After eighth 24 h, KT Lymphatic application on the lower leg.

In the patients of the research group, in the third 24 h after the surgery, KT 10 mm dynamic tapes in the shape of a fan were applied to the lateral and medial sides of the tibia. The lymphatic drainage technique was introduced. The proximal point, the tip of the tape, i.e., the anchor of the tape, was applied without tension at the level of the popliteal fossa in the proximity of the fibula head lymph nodes on the medial side of a knee. Distal points, or tails, were applied with 15% tension and reached the lateral ankle and medial tibia (Figure 2). The tapes remained on the skin for 5 consecutive days [24,25]. On the eighth 24 h, they were removed, and the skin was cleansed (Figure 3). The application of the tapes was carried out by a qualified physiotherapist with 20-years of experience in rehabilitating patients.

#### 2.1.4. ROM Assessment in a Knee Joint

On the third and the eighth 24 h after the surgery, the ROM in the knee joint was tested by the use of a goniometer. The initial position was lying down with straight lower limbs. The goniometer axis was set in the proximity of the fibula head in compliance with the transverse axis of the joint. The fixed arm of the goniometer was aimed at the greater trochanter of the femur, and the mobile arm was aimed at the lateral malleolus [26] (Figure 1).

#### 2.1.5. Postoperational Rehabilitation Programme

The rehabilitation program for all of the 82 patients was equal and introduced in the first 24 h after the surgery. The program included exercises to prevent blood clotting in the lower limbs, inner range quads exercises, isometric gluteus and lower leg exercises, diaphragm breathing exercises, and active slow exercises.

The training was conducted with the application of a passive movement for flexion and extension in the knee joint with the use of CPM within a tolerated painless ROM [27,28,29]. In the second 24 h after the surgery, the patients were actively verticalized with the use of orthopedic appliances, such as walking frames. In the second or third 24 h, the drains were removed. In both groups, a cold pack compress was applied for the first 24 h after the surgery in order to reduce postoperational edema and pain [28,30,31] (Figure 1).

### 2.2. Statistical Analysis

In order to present the results in categorical and ordinal scales, methods of descriptive statistics were introduced, i.e., number (N) and percentage (%). The statistical analysis included the test of independence, χ^2^, in order to assess the relationship between the examined variables on categorical and ordinal scales. In order to present the results on a quantitative scale, the following methods of descriptive statistics were introduced: arithmetic mean (x), median (Me), standard deviation (SD), minimum (Min), maximum (Max), and interquartile range (IQR). In order to assess the compliance of the distribution of the examined variables with normal distribution, a Shapiro–Wilk test was introduced. In the cases where there was no normal distribution of variables, nonparametric tests were applied, and, in the cases when a normal distribution of variables was revealed, parametric tests were applied. Wilcoxon’s signed-ranked test was applied in order to assess the difference between the two measurements. The Student’s t-test was applied for the independent trials, and the Mann–Whitney test was applied in order to assess the differences in the values of the variables between the two groups. The assessment of the correlation between the variables was defined by Pearson correlation and Spearman’s rank correlation. Statistical inference of 5% and related to its level of statistical significance (α) of 0.05 (α = 0.05) were assumed. On the basis of the results of the analysis, the probability value rules were applied: *p* < 0.05—statistical significance, *p* < 0.01 strong statistical significance, and *p* < 0.001—very strong statistical significance. The statistical analysis was conducted by Statistica v.13.0 software (StatSoft, Tulsa, OK, USA). In order to collect and support statistical analyses, MS Excel 2010 (Microsoft, Redmond, WA, USA) software was used.

## 3. Results

### 3.1. Comparison of Differences in Linear Measurement in Ultrasound Scan between ‘KT’ Group and ‘Non-KT’ Group

The examination revealed statistically a significant difference in the linear measurements above the fibula head at its peak in patients with KT compared to patients without KT (t = −3.01; *p* < 0.01). In the group of patients with KT, a mean decrease of −1.12 mm was observed, whereas in the group of patients without KT, a mean decrease of −0.18 mm was observed (Table 2).

The research revealed a statistically significant difference in the linear measurements of 2.5 cm from the fibula neck in the group of patients with KT compared to the patients without KT (t = −2.60; *p* < 0.05). In the group of patients with KT, a mean decrease of −0.9 mm was observed, whereas in the group of patients without KT, a mean increase of 0.02 mm was observed (Table 3).

The research revealed a statistically significant difference in the linear measurement of 5 cm from the fibula neck in the group of patients with KT compared to the patients without KT (Z = −2.64; *p* < 0.01). In the group of patients with KT, a mean decrease of −0.82 mm was observed, whereas in the group of patients without KT, the mean value of the change was 0 mm (Table 4).

### 3.2. Comparison of Differences in Knee Flexion Angle in the Third and the Eighth 24 Hours in ‘KT’ Group and ‘Non-KT’ Group

The research revealed a statistically significant greater knee flexion angle in the patients with KT (Z = 5.51; *p* < 0.001). The research revealed a statistically significant greater knee flexion angle in patients without KT (Z = 5.37; *p* < 0.001) (Table 5).

The research did not reveal any statistically significant differences in knee flexion angle in patients with KT compared to patients without KT (t = 1.55; *p* > 0.05) (Table 6).

The research revealed no statistically significant correlation between the change in ROM in a knee joint and the change of parameters specific to the subcutaneous edema in the group of patients without KT (Table 7).

## 4. Discussion

The aim of this work was to determine the effect of Kinesio Taping (KT) in reducing edema of the subcutaneous tissue and improving the ROM in the joint. In our study, we saw a positive effect of KT on the amount of swelling. This involves the tape being stuck to the skin to gently lift it, thus removing lymphatic obstructions and reducing pressure by widening the space between the dermis and fascia. It causes an uninhibited flow of lymph and blood. This mechanism results in accelerated tissue healing when lymphatic obstruction and edema are reduced [15,32,33]. In addition, skin elevation elongates the interstitial space, which reduces pressure on subcutaneous nociceptors [33,34,35]. Previous studies highlight the positive effects of KT on lymphedema [36,37].

Tsai et al. [38] compared traditional arm dressing after breast cancer surgery to the lymphatic drainage application of dynamic tape. In their research paper, the authors stated that in patients with KT, the swelling was reduced faster and with better acceptance than in patients with elastic bandages applied for longer periods [38]. Another example of lymphatic drainage application in postoperational facial skeleton edema was presented by Lietz-Kijak et al. in their research paper. The authors carried out a pilot experiment on 16 patients after orthognathic surgery in which KT was applied twice with a two-day interval in the facial area. The research revealed a statistically significant reduction in edema, which had a substantial influence on further treatment and facial functions [39]. Similar research regarding the efficacy of the lymphatic drainage KT technique after temporomandibular joint surgeries was carried out on a group of 30 patients by Ristow [40]. The aim of his research was to establish if the application of dynamic tape prevents edema, pain, and trismus after the surgery of a broken zygomatic bone and if it improves postoperational quality of life. As in the research of my own, the KT was applied once in the first days after the surgery for a period of 5 days. The growth of facial edema was assessed with linear measurements, and a difference of 60% was revealed compared to a control group in the first two days of application. In the abovementioned research, the KT application had no influence on pain sensation and the ROM in the temporomandibular joints [40,41]. The influence of dynamic KT tapes on postoperational edema was also examined by Białoszewski et al. [42] in patients treated with the Lizarow method in the lower limbs. In their paper, the authors compared the effectiveness of lymphatic drainage application to the application of traditional lymphatic drainage massage.

The application of taping in a sample group resulted in a greater reduction in edema and the reduction in hip and lower leg circumference than after the application of lymphatic drainage [42]. As in the research of my own, V. Donec et al. [43] assessed the influence of anti-swelling KT and other physiotherapeutical treatments used routinely on the reduction in postoperational edema, pain, and ROM improvement after the primary endoprosthetics of a knee joint. The sample group consisted of 94 patients. In all of them, significant improvement was observed regarding pain sensation, measured with a numeric pain rating scale, NPRS, and a statistically significant reduction in limb circumference in patients with KT was revealed. The technique applied in Donec’s research had no significant influence on ROM in a knee joint in either of the groups [43]. The abovementioned study revealed results that indicate the positive influence of anti-swelling properties and edema reduction (t = −3.01; *p* < 0.05). In Donec’s research, KT was also applied on the second 24 h after the surgery and remained attached for up to 28 days, re-applied every fifth day with a one-day interval. Our results are consistent with those of Jarecki, based on a smaller group [20]. In the research of my own, in addition to a centimeter tape measure, an ultrasound scan was also applied, which enabled an objective evaluation of the results and a detailed measure of the subcutaneous tissue. Ultrasound imaging facilitates a detailed assessment of soft tissues surrounding the joint statically and dynamically [44]. Summing up, postoperational subcutaneous edema is a considerable problem in the initial phase of physiotherapy of patients after surgeries. KT dynamic taping is a reliable alternative method, and it supports edema treatment. It should be considered for ongoing therapy for patients with surgical edema. It may accelerate the comfort of hospitalization and surgical sequelae. It is advised to carry out further research in order to prove the effectiveness of the KT method in improving ROM. It should cover a larger number of patients and be extended by the application of dynamic taping after other surgeries in order to enable an objective evaluation of the results of the treatment.

The study presented here has several limitations. The first limitation is the evaluation of therapeutic effects for up to 8 days. Future studies should increase the time to observe treatment effects in subjects. The time of 8 days was related to the average time of patients’ discharge from the hospital. This is a crucial time to implement rehabilitation management. Another limitation was the lack of use of an adapted activity protocol, which should be considered in future studies. Another limitation was the lack of consideration of the patients’ lifestyle habits before surgery, e.g., alcohol consumption and cigarette smoking.

## 5. Conclusions

The results indicate that the spread of subcutaneous tissue edema after KT application was significantly reduced, so the extension of postoperative physiotherapy to include KT lymphatic application should be considered. Further research into the possible effects of lymphatic KT on ROM is suggested.

## Figures and Tables

**Table 1:** Patients characteristics.

	KT	Without KT	Test		*p*
*n*	42	40	-	-	-
Age (years)	66.69 ± 6.78	67.78 ± 7.56	T	−0.68	0.51
Sex	M = 33 K = 9	M = 32 F = 8	Z	0.11	0.92
Weight (kg)	81.62 ± 13.57	80.05 ± 15.15	T	0.49	0.62
Height (m)	164.93 ± 6.87	163.20 ± 8.76	Z	1.21	0.23
BMI	30.03 ± 4.85	30.10 ± 5.59	T	−0.06	0.95

**Table 2:** Differences [mm] in linear measurement taken above fibula head at the highest point.

	Group	*n*	x	Me	Min	Max	IQR	SD	Student’s *t*-Test	
									T	*p*
Differences in mm	KT	42	−1.12	−1.10	−4.50	1.70	1.90	1.32	−3.01	<0.01 *
Differences in mm	Without KT	40	−0.18	0.00	−3.40	2.70	1.70	1.51		

* Significant difference, IQR—interquartile range, SD—standard deviation, KT—Kinesio Taping.

**Table 3:** Differences [mm] in linear measurement taken 2.5 cm above fibula neck.

	Group	*n*	x	Me	Min	Max	IQR	SD	Student’s *t*-Test	
									T	*p*
Difference in mm	KT	42	−0.90	−0.75	−3.90	2.90	1.40	1.43	−2.60	<0.05 *
Difference in mm	Without KT	40	0.02	−0.05	−4.20	4.40	1.85	1.76		

* Significant difference, IQR—interquartile range, SD—standard deviation, KT—Kinesio Taping.

**Table 4:** Differences [mm] in linear measurement taken 5 cm above fibula neck.

	Group	*n*	x	Me	Min	Max	IQR	SD	Mann–Whitney Test	
									Z	*p*
Difference in mm	KT	42	−0.82	−0.80	−3.60	3.20	1.40	1.34	−2.64	<0.01 *
Difference in mm	Without KT	40	0.00	0.00	−5.20	3.40	2.00	1.79		

* Significant difference, IQR—interquartile range, SD—standard deviation, KT—Kinesio Taping.

**Table 5:** Measurement of the knee joint angle.

	Group	*n*	x	Me	Min	Max	IQR	SD	Wilcoxon Test	
									Z	*p*
3rd 24 h without KT; flexion angle	With KT before	42	44.48	40.00	20.00	80.00	12.00	13.40	5.51	<0.001 *
8th 24 h after KT; flexion angle	With KT after	42	68.64	70.00	40.00	110.00	20.00	15.52		
3rd 24 h without KT; flexion angle	Without KT before	40	41.63	40.00	20.00	75.00	13.50	12.01	5.37	<0.001 *
8th 24 h after KT; flexion angle	Without KT after	40	61.73	60.00	30.00	95.00	20.00	15.97		

* Significant difference, IQR—interquartile range, SD—standard deviation, KT—Kinesio Taping.

**Table 6:** Difference in knee flexion angle.

	Group	*n*	x	Me	Min	Max	IQR	SD	Student’s *t*-Test	
									T	*p*
Difference flexion angle	KT	42	24.17	20.00	0.00	60.00	15.00	13.02	1.55	>0.05

IQR—interquartile range, SD—standard deviation, KT—Kinesio Taping.

**Table 7:** The results of the correlation analysis of the change in improvement in the range of motion (ROM) in the joint with the variables characterizing the level of subcutaneous tissue swelling according to the Spearman and Pearson correlation.

Variable	Group	*n*	R	*p*
Difference in mm	KT	42	0.02	>0.05
	without KT	40	0.01	>0.05
Difference in mm	KT	42	0.05	>0.05
	Without KT	40	0.01	>0.05
Difference in mm	KT	42	−0.18	>0.05
	Without KT	40	0.03	>0.05
Circuit difference at the height of the fibula head (cm)	KT	42	−0.22	>0.05
	Without KT	40	−0.11	>0.05
Circuit difference 2.5 cm above fibula neck (cm)	KT	42	−0.08	>0.05
	Without KT	40	−0.06	>0.05
Circuit difference 5 cm above fibula neck (cm)	KT	42	−0.17	>0.05
	Without KT	40	0.09	>0.05

KT—Kinesio Taping.

## Data Availability

Not applicable.

## References

[1] Sen R., Hurley J.A. (2022). Osteoarthritis. StatPearls.

[2] Chen D., Shen J., Zhao W., Wang T., Han L., Hamilton J.L., Im H.-J. (2017). Osteoarthritis: Toward a Comprehensive Understanding of Pathological Mechanism. Bone Res..

[3] Castorina S., Guglielmino C., Castrogiovanni P., Szychlinska M.A., Ioppolo F., Massimino P., Leonardi P., Maci C., Iannuzzi M., Di Giunta A. (2019). Clinical Evidence of Traditional vs Fast Track Recovery Methodologies after Total Arthroplasty for Osteoarthritic Knee Treatment. A Retrospective Observational Study. Muscle Ligaments Tendons J..

[4] Felson D.T. (2004). An Update on the Pathogenesis and Epidemiology of Osteoarthritis. Radiol. Clin. N. Am..

[5] Altman R., Asch E., Bloch D., Bole G., Borenstein D., Brandt K., Christy W., Cooke T.D., Greenwald R., Hochberg M. (1986). Development of Criteria for the Classification and Reporting of Osteoarthritis: Classification of Osteoarthritis of the Knee. Arthritis Rheum..

[6] Page C.J., Hinman R.S., Bennell K.L. (2011). Physiotherapy Management of Knee Osteoarthritis. Int. J. Rheum. Dis..

[7] Carr A.J., Robertsson O., Graves S., Price A.J., Arden N.K., Judge A., Beard D.J. (2012). Knee Replacement. Lancet.

[8] Felson D.T., Lawrence R.C., Dieppe P.A., Hirsch R., Helmick C.G., Jordan J.M., Kington R.S., Lane N.E., Nevitt M.C., Zhang Y. (2000). Osteoarthritis: New Insights. Part 1: The Disease and Its Risk Factors. Ann. Intern. Med..

[9] Jordan K.M., Arden N.K., Doherty M., Bannwarth B., Bijlsma J.W.J., Dieppe P., Gunther K., Hauselmann H., Herrero-Beaumont G., Kaklamanis P. (2003). EULAR Recommendations 2003: An Evidence Based Approach to the Management of Knee Osteoarthritis: Report of a Task Force of the Standing Committee for International Clinical Studies Including Therapeutic Trials (ESCISIT). Ann. Rheum. Dis..

[10] Lim W.B., Al-Dadah O. (2022). Conservative Treatment of Knee Osteoarthritis: A Review of the Literature. World J. Orthop..

[11] Hunter D.J., Lo G.H. (2008). The Management of Osteoarthritis: An Overview and Call to Appropriate Conservative Treatment. Rheum. Dis. Clin. N. Am..

[12] Picard F., Deakin A., Balasubramanian N., Gregori A. (2018). Minimally Invasive Total Knee Replacement: Techniques and Results. Eur. J. Orthop. Surg. Traumatol. Orthop. Traumatol..

[13] Williamson L., Wyatt M.R., Yein K., Melton J.T.K. (2007). Severe Knee Osteoarthritis: A Randomized Controlled Trial of Acupuncture, Physiotherapy (Supervised Exercise) and Standard Management for Patients Awaiting Knee Replacement. Rheumatol. Oxf. Engl..

[14] Russell T.G., Buttrum P., Wootton R., Jull G.A. (2011). Internet-Based Outpatient Telerehabilitation for Patients Following Total Knee Arthroplasty: A Randomized Controlled Trial. J. Bone Jt. Surg. Am..

[15] Kase K., Wallis J., Tsuyoshi K. (2013). Clinical Therapeutic Applications of the Kinesio Taping Method.

[16] Kase K., Hashimoto T., Tomoki O. (1996). Development of Kinesio Tape. Kinesio Taping Perfect Manual.

[17] Castrogiovanni P., Di Giunta A., Guglielmino C., Roggio F., Romeo D., Fidone F., Imbesi R., Loreto C., Castorina S., Musumeci G. (2016). The Effects of Exercise and Kinesio Tape on Physical Limitations in Patients with Knee Osteoarthritis. J. Funct. Morphol. Kinesiol..

[18] Krajczy M., Luniewski J., Bogacz K., Dybek T., Kiczyński P., Krajczy E., Szczegielniak A., Szczegielniak J. (2014). Impact of Elastic Therapeutic Tape on Final Effects of Physiotherapy in Patients with Colles’ Fracture. Fizjoterapia Pol..

[19] Mostafavifar M., Wertz J., Borchers J. (2012). A Systematic Review of the Effectiveness of Kinesio Taping for Musculoskeletal Injury. Phys. Sportsmed..

[20] Jarecki J., Sobiech M., Turżańska K., Tomczyk-Warunek A., Jabłoński M. (2021). A Kinesio Taping Method Applied in the Treatment of Postsurgical Knee Swelling after Primary Total Knee Arthroplasty. J. Clin. Med..

[21] Hörmann J., Vach W., Jakob M., Seghers S., Saxer F. (2020). Kinesiotaping for Postoperative Oedema—What Is the Evidence? A Systematic Review. BMC Sports Sci. Med. Rehabil..

[22] Kohn M.D., Sassoon A.A., Fernando N.D. (2016). Classifications in Brief: Kellgren-Lawrence Classification of Osteoarthritis. Clin. Orthop..

[23] Schiphof D., de Klerk B.M., Kerkhof H.J.M., Hofman A., Koes B.W., Boers M., Bierma-Zeinstra S.M.A. (2011). Impact of Different Descriptions of the Kellgren and Lawrence Classification Criteria on the Diagnosis of Knee Osteoarthritis. Ann. Rheum. Dis..

[24] Tiffert M. (2010). Kinesiology Taping-Teoria, Metodyka, Przykładowe Aplikacje w Konkretnych Dysfunkcjach. Prakt. Fizjoterapia Rehabil..

[25] Hałas I. (2010). Kinesiology Taping Metoda Wspomagająca Teratpię Tkanek Miękkich. Praktyczna Fizjoterapia i Rehabilitacja.

[26] Boone D.C., Azen S.P., Lin C.M., Spence C., Baron C., Lee L. (1978). Reliability of Goniometric Measurements. Phys. Ther..

[27] Artz N., Elvers K.T., Lowe C.M., Sackley C., Jepson P., Beswick A.D. (2015). Effectiveness of Physiotherapy Exercise Following Total Knee Replacement: Systematic Review and Meta-Analysis. BMC Musculoskelet. Disord..

[28] Denis M., Moffet H., Caron F., Ouellet D., Paquet J., Nolet L. (2006). Effectiveness of Continuous Passive Motion and Conventional Physical Therapy after Total Knee Arthroplasty: A Randomized Clinical Trial. Phys. Ther..

[29] Herbold J.A., Bonistall K., Blackburn M., Agolli J., Gaston S., Gross C., Kuta A., Babyar S. (2014). Randomized Controlled Trial of the Effectiveness of Continuous Passive Motion after Total Knee Replacement. Arch. Phys. Med. Rehabil..

[30] Minns Lowe C., Barker K., Dewey M., Sackley C. (2007). Effectiveness of Physiotherapy Exercise after Knee Arthroplasty for Osteoarthritis: Systematic Review and Meta-Analysis of Randomised Controlled Trials. BMJ.

[31] Chughtai M., Sodhi N., Jawad M., Newman J.M., Khlopas A., Bhave A., Mont M.A. (2017). Cryotherapy Treatment After Unicompartmental and Total Knee Arthroplasty: A Review. J. Arthroplast..

[32] Dębska M. (2015). Kinesiology Taping Jako Metoda Terapeutyczna I Kosmetyczna W Stłuczeniu Mięśnia—Opis Przypadku. Polski Przegląd Nauk o Zdrowiu.

[33] Osorio J.A., Vairo G.L., Rozea G.D., Bosha P.J., Millard R.L., Aukerman D.F., Sebastianelli W.J. (2013). The Effects of Two Therapeutic Patellofemoral Taping Techniques on Strength, Endurance, and Pain Responses. Phys. Ther. Sport Off. J. Assoc. Chart. Physiother. Sports Med..

[34] Murray H.M. (2000). Effects of Kinesio Taping^®^ on Muscle Strength after ACL-Repair. J. Orthop. Sports Phys. Ther..

[35] Kahanov L. (2007). Kinesio Taping^®^, Part 1: An Overview of Its Use in Athletes. Athl. Ther. Today.

[36] Tantawy S.A., Abdelbasset W.K., Nambi G., Kamel D.M. (2019). Comparative Study between the Effects of Kinesio Taping and Pressure Garment on Secondary Upper Extremity Lymphedema and Quality of Life Following Mastectomy: A Randomized Controlled Trial. Integr. Cancer Ther..

[37] Bosman J., Piller N. (2010). Lymph Taping and Seroma Formation Post Breast Cancer. J. Lymphoedema.

[38] Tsai H.-J., Hung H.-C., Yang J.-L., Huang C.-S., Tsauo J.-Y. (2009). Could Kinesio Tape Replace the Bandage in Decongestive Lymphatic Therapy for Breast-Cancer-Related Lymphedema? A Pilot Study. Support. Care Cancer.

[39] Lietz-Kijak D., Kijak E., Krajczy M., Bogacz K., Łuniewski J., Szczegielniak J. (2018). The Impact of the Use of Kinesio Taping Method on the Reduction of Swelling in Patients After Orthognathic Surgery: A Pilot Study. Med. Sci. Monit..

[40] Ristow O., Pautke C., Kehl V., Koerdt S., Schwärzler K., Hahnefeld L., Hohlweg-Majert B. (2014). Influence of Kinesiologic Tape on Postoperative Swelling, Pain and Trismus after Zygomatico-Orbital Fractures. J. Cranio-Maxillofac. Surg..

[41] Ristow O., Hohlweg-Majert B., Kehl V., Koerdt S., Hahnefeld L., Pautke C. (2013). Does Elastic Therapeutic Tape Reduce Postoperative Swelling, Pain, and Trismus after Open Reduction and Internal Fixation of Mandibular Fractures?. J. Oral Maxillofac. Surg..

[42] Białoszewski D., Woźniak W., Zarek S. (2009). Clinical Efficacy of Kinesiology Taping in Reducing Edema of the Lower Limbs in Patients Treated with the Ilizarov Method—Preliminary Report. Ortop. Traumatol. Rehabil..

[43] Donec V., Kriščiūnas A. (2014). The Effectiveness of Kinesio Taping^®^ after Total Knee Replacement in Early Postoperative Rehabilitation Period. A Randomized Controlled Trial. Eur. J. Phys. Rehabil. Med..

[44] Hassan S. (2018). Overview of Musculoskeletal Ultrasound for the Clinical Rheumatologist. Clin. Exp. Rheumatol..

